# Supplementary material for: Analysis of the Economic Burden of COVID-19 on the Workers of a Teaching Hospital in the Centre of Italy: Changes in Productivity Loss and Healthcare Costs Pre and Post Vaccination Campaign
Source: Vaccines (Basel). 2023 Nov 30;11(12):1791. doi: 10.3390/vaccines11121791 (PMC10747819; doi:10.3390/vaccines11121791)
Supplement: Supplementary file 1 [file vaccines-11-01791-s001.zip › vaccines-2657918-supplementary.pdf]

## Annex A.1 – Categories of hospital workers

| Categories (ITA) | Code | Categories (EN) |
|---|---|---|
| **Assistente sanitario** | **A000** | Healthcare assistant |
| Dirigente di struttura complessa e Direttore sanitario | A001 | Healthcare executive |
| Caposala | A002 | Head nurse |
| Coordinatore infermieristico | A003 | Nursing coordinator |
| Infermiere | A004 | Nurse |
| Infermiere in Cooperativa | A005 | Nurse (Cooperativa) |
| Collaboratore sanitario | A007 | Healthcare worker |
| Coordinatore ostetrica | A008 | Obstetric coordinator |
| Ostetrico | A009 | Obstetrician |
| Dietista | A010 | Dietician |
| Direttore Ortottica | A011 | Orthoptic director |
| Ortottista e assistente di oftalmologia | A012 | Orthoptist and assistant of ophthalmology |
| Fisioterapista | A013 | Physiotherapist |
| Igienista dentale | A014 | Dental hygienist |
| Logopedista | A015 | Speech therapist |
| Podologo | A016 | Podiatrist |
| Psicologo | A017 | Psychologist |
| Anestesista | A018 | Anesthetist |
| Direttore Unità Operativa Complessa (UOC) - Medico | A019 | Complex Operational Unit Director (COU) - Medical |
| Dirigente medico | A020 | Medical director |
| Medico | A021 | Medical doctor (MD) |
| Responsabile di Area Dipartimentale (RAD) | A022 | Departmental Area Manager |
| **Tecnico** | **B000** | **Technician** |
| Dirigente tecnico | B001 | Technical manager |
| Assistente tecnico | B002 | Technical assistant |
| Coordinatore di area riabilitativa e tecnico-sanitaria | B003 | Technical coordinator |
| Operatore tecnico specializzato | B004 | Specialised technical operator |
| Odontotecnico | B005 | Dental technician |
| Capo servizio Tecnico sanitario di radiologia medica (TSRM) | B006 | Head of Technical Health Service of Medical Radiology (THSMR) |
| Coordinatore Tecnico sanitario di radiologia medica (TSRM) | B007 | Coordinator of Technical Health Service of Medical Radiology (THSMR) |

| | | |
|---|---|---|
| Tecnico sanitario di radiologia medica (TSRM) | B008 | Radiologist technician |
| Tecnico audiometrista | B009 | Audiometrist technician |
| Tecnico audioprotesista | B010 | Hearing aid technician |
| Tecnico della prevenzione nell'ambiente e nei luoghi di lavoro | B011 | Prevention technician in the environment and in the workplace |
| Tecnico dell'educazione e riabilitazione psichiatrica e psicosociale | B012 | Psychiatric rehabilitation technician |
| Tecnico di neurofisiopatologia | B013 | Neurophysiopathology technician |
| Tecnico di fisiopatologia cardiocircolatoria e perfusione cardiovascolare | B014 | Cardiocirculatory physiopathology and cardiovascular perfusion technician |
| Tecnico ortopedico | B015 | Orthopedic technician |
| Tecnico sanitario di laboratorio biomedico | B016 | Biomedical laboratory health technician |
| Terapista della neuro e psicomotricità dell'età evolutiva | B017 | Neuro and psychomotor therapist of the developmental age |
| Terapista occupazionale | B018 | Occupational therapist |
| **Operatore socio sanitario** | **C000** | **Social health operator** |
| Operatore socio sanitario - Cooperativa | C001 | Social health operator - Cooperative |
| Ausiliario socio-sanitario | C002 | Healthcare auxiiliary |
| Ausiliario socio-sanitario - Cooperativa | C003 | Healthcare auxiiliary - Cooperative |
| Assistente sociale | C004 | Social worker |
| Operatore socio assistenziale (OSA) | C005 | Social welfare worker |
| Puericultrice | C006 | Social child health operator |
| Vigilatrice infanzia | C007 | Childhood vigilante |
| Personale supporto | C008 | Support personnel |
| **Farmacista** | **D000** | **Pharmacist** |
| Operatore farmacia | D001 | Pharmacy operator |
| **Impiegato amministrativo** | **E000** | **Administrative** |
| Assistente amministrativo | E001 | Administrative assistant |
| Dirigente amministrativo | E002 | Managing director |
| Direttore Unità Operativa Complessa (UOC) - Non medico | E003 | Complex Operational Unit Director (COU) - Not medical |
| **Support services** | **F000** | **Support services** |
| Addetto pulizie | F001 | Cleaning attendant |
| Addetto mensa | F002 | Canteen clerk |
| Cuoco | F003 | Cook |
| Addetto Guardaroba | F004 | Cloakroom attendant |
| Antincendio | F005 | Fire fighting |
| Archivista | F006 | Archivist |

| | | |
|---|---|---|
| Autista | F007 | Driver |
| Operatore autoparco | F008 | Fleet operator |
| Referente autoparco | F009 | Parking contact person |
| Bibliotecaria | F010 | Libriarian |
| Cappellano | F011 | Chaplain |
| Coordinatore magazzino | F012 | Warehouse coordinator |
| Magazziniere | F013 | Warehouse operator |
| COVID-19 case manager | F014 | COVID-19 case manager |
| COVID-19 case tracer | F015 | COVID-19 case tracer |
| Ingegnere gestionale | F016 | Management engineer |
| Ingegnere biomedico | F017 | Biomedical engineer |
| ICT technician | F018 | ICT technician |
| Manutenzione | F019 | Maintenance |
| Security | F020 | Security |
| Servizio di accoglienza in DEA | F021 | Reception service in DEA |
| Volunteer | F022 | Volunteer |
| Insegnante | F023 | Teacher |
| Biologo | F024 | Biologist |
| **Student (incl. Training students from in all the departments of the faculty)** | **G000** | **Student (incl. Training students from in all the departments of the faculty)** |
| PhD Student | G001 | PhD Student |
| Resident | G002 | Resident |
| Professor (include Associate Professor) | G003 | Professor (include Associate Professor) |
| **Researcher** | **H000** | **Researcher** |
| Assegnista di ricerca | H001 | Research fellow |
| Responsabile universitario | H002 | University manager |

## Annex A.2 – Integrated Care departments of the hospital

| Dipartimenti Assistenziali Integrati (IT) | Integrated Care Department (EN) |
|---|---|
| Materno Infantile e Science UroGinecologiche | Maternal Infantile and Urogynecological Science |
| Chirurgia Generale, Chirurgia Plastica e Ortopedia | General Surgery, Plastic Surgery and Orthopedics |
| Ambulatorio Polispecialistico | Multi-specialist outpatient clinic |
| Amministrazione | Administration |
| Support services | Support services |
| Dipartimento Tecnico-Amministrativo | Technical-Administrative Department |
| Medicina Diagnostica e Radiologia | Diagnostic Medicine and Radiology |
| Emergenza-Accettazione, Aree Critiche e Trauma | Emergency-Acceptance, Critical Areas and Trauma |
| Testa Collo | Head Neck |
| Cardio-Toraco-Vascolare e Chirurgia dei Trapianti d'Organo | Cardio-Thoraco-Vascular and Organ Transplant Surgery |
| Dipartimento Organizzazione Ospedaliera | Hospital Organization Department |
| Chirurgia | Surgery |
| Chirurgia - COVID | Surgery - COVID |
| Cuore e Grossi Vasi | Heart and Big Vases |
| Medicina Interna e Specialità Mediche | Internal Medicine and Medical Specialties |
| Ematologia, Oncologia e Dermatologia | Hematology, Oncology and Dermatology |
| Medicina Interna, Science Endocrino-metaboliche e Malattie Infettive | Internal Medicine, Endocrine-Metabolic Science and Infectious Diseases |
| Direzione strategica | Strategic Direction |
| Dipartimento Professioni Sanitarie | Department of Health Professions |
| Hub vaccinale | Vaccination Hub |
| Ufficio Affari Legali e Contenzioso | Legal Affairs and Litigation Office |
| Medicina Interna | Internal Medicine |
| Medicina Sperimentale | Experimental Medicine |
| Medicina Interna - COVID | Internal Medicine - COVID |
| Neuroscience e Salute Mentale | Neuroscience and Mental Health |
| Terapia Intensiva COVID | COVID Intensive Care |
| Università | University |
| Ufficio Protocollo | Protocol Office |

## Annex B – Gross hourly salary associated with hospital role and source of information

| Code | Role EN | Gross Hourly salary | Source |
|---|---|---|---|
| **A000** | Healthcare assistant | € 11.53 | CCNL |
| **A001** | Healthcare executive | € 29.36 | CCNL |
| **A002** | Head nurse | € 12.13 | CCNL |
| **A003** | Nursing coordinator | € 12.13 | CCNL |
| **A004** | Nurse | € 11.53 | CCNL |
| **A005** | Nurse (Cooperativa) | € 9.22 | CCNL |
| **A007** | Healthcare worker | € 10.62 | CCNL |
| **A008** | Obstetric coordinator | € 12.13 | CCNL |
| **A009** | Obstetrician | € 11.53 | CCNL |
| **A010** | Dietician | € 11.53 | CCNL |
| **A011** | Orthoptic director | € 11.53 | CCNL |
| **A012** | Orthoptist and assistant of ophthalmology | € 11.53 | CCNL |
| **A013** | Physiotherapist | € 11.53 | CCNL |
| **A014** | Dental hygienist | € 11.53 | CCNL |
| **A015** | Speech therapist | € 11.53 | CCNL |
| **A016** | Podiatrist | € 11.53 | CCNL |
| **A017** | Psychologist | € 21.50 | Job portal |
| **A018** | Anesthetist | € 36.68 | Job portal |
| **A019** | Complex Operational Unit Director (COU) - Medical | € 29.36 | CCNL |
| **A020** | Medical director | € 29.36 | CCNL |
| **A021** | Medical doctor (MD) | € 21.77 | Interview |
| **A022** | Departmental Area Manager | € 29.36 | CCNL |
| **B000** | Technician | € 16.36 | Interview |
| **B001** | Technical manager | € 11.53 | CCNL |
| **B002** | Technical assistant | € 10.94 | CCNL |
| **B003** | Technical coordinator | € 12.13 | CCNL |
| **B004** | Specialised technical operator | € 10.94 | CCNL |
| **B005** | Dental technician | € 11.53 | CCNL |
| **B006** | Head of Technical Health Service of Medical Radiology (THSMR) | € 11.53 | CCNL |
| **B007** | Coordinator of Technical Health Service of Medical Radiology (THSMR) | € 11.53 | CCNL |
| **B008** | Radiologist technician | € 16.36 | Interview |
| **B009** | Audiometrist technician | € 11.53 | CCNL |
| **B010** | Hearing aid technician | € 11.53 | CCNL |
| **B011** | Prevention technician in the environment and in the workplace | € 11.53 | CCNL |
| **B012** | Psychiatric rehabilitation technician | € 11.53 | CCNL |
| **B013** | Neurophysiopathology technician | € 11.53 | CCNL |
| **B014** | Cardiocirculatory physiopathology and cardiovascular perfusion technician | € 11.53 | CCNL |
| **B015** | Orthopedic technician | € 11.53 | CCNL |
| **B016** | Biomedical laboratory health technician | € 16.36 | Interview |
| **B017** | Neuro and psychomotor therapist of the developmental age | € 11.53 | CCNL |
| **B018** | Occupational therapist | € 11.53 | CCNL |
| **C000** | Social health operator | € 10.62 | CCNL |

| | | | |
|---|---|---|---|
| **C001** | Social health operator - Cooperative | € 8.49 | CCNL |
| **C002** | Healthcare auxiiliary | € 9.77 | CCNL |
| **C003** | Healthcare auxiiliary - Cooperative | € 7.81 | CCNL |
| **C004** | Social worker | € 11.53 | CCNL |
| **C005** | Social welfare worker | € 9.77 | CCNL |
| **C006** | Social child health operator | € 10.94 | CCNL |
| **C007** | Childhood vigilante | € 10.62 | CCNL |
| **C008** | Support personnel | € 9.77 | CCNL |
| **D000** | Pharmacist | € 21.35 | Job portal |
| **D001** | Pharmacy operator | € 20.30 | Job portal |
| **E000** | Administrative | € 15.30 | Interview |
| **E001** | Administrative assistant | € 11.53 | CCNL |
| **E002** | Managing director | € 20.92 | Job portal |
| **E003** | Complex Operational Unit Director (COU) - Not medical | € 29.36 | CCNL |
| **F000** | Support services | € 11.53 | CCNL |
| **F001** | Cleaning attendant | € 9.77 | CCNL |
| **F002** | Canteen clerk | € 9.77 | CCNL |
| **F003** | Cook | € 10.62 | CCNL |
| **F004** | Cloakroom attendant | € 9.77 | CCNL |
| **F005** | Fire fighting | € 18.21 | Interview |
| **F006** | Archivist | € 11.76 | Job portal |
| **F007** | Driver | € 10.62 | CCNL |
| **F008** | Fleet operator | € 10.62 | CCNL |
| **F009** | Parking contact person | € 10.62 | CCNL |
| **F010** | Libriarian | € 8.71 | Job portal |
| **F011** | Chaplain | € 11.53 | CCNL |
| **F012** | Warehouse coordinator | € 14.72 | Job portal |
| **F013** | Warehouse operator | € 11.33 | Job portal |
| **F014** | COVID-19 case manager | € 29.36 | CCNL |
| **F015** | COVID-19 case tracer | € 29.36 | CCNL |
| **F016** | Management engineer | € 16.55 | Job portal |
| **F017** | Biomedical engineer | € 16.82 | Job portal |
| **F018** | ICT technician | € 10.94 | CCNL |
| **F019** | Maintenance | € 10.62 | CCNL |
| **F020** | Security | € 10.94 | CCNL |
| **F021** | Reception service in DEA | € 10.94 | CCNL |
| **F022** | Volunteer | € 0.00 | - |
| **F023** | Teacher | € 10.62 | CCNL |
| **F024** | Biologist | € 15.68 | Job portal |
| **G000** | Student (incl. Training students from in all the departments of the faculty) | € 0.00 | - |
| **G001** | PhD Student | € 8.41 | Job portal |
| **G002** | Resident | € 12.45 | Job portal |
| **G003** | Professor (include Associate Professor) | € 24.83 | Interview |
| **H000** | Researcher | € 13.94 | Job portal |
| **H001** | Research fellow | € 13.94 | Job portal |
